# Predicting functional movement capacity in adults: the effect of range of motion and isometric strength

**DOI:** 10.1186/s13102-024-00935-0

**Published:** 2024-07-02

**Authors:** Mazhar Özkan, Umut Canli, Reem Alwhaibi, Kübra Ustaömer, Aydın Karaçam, Bekir Erhan Orhan, Lucimere Bohn, Kenan Sivrikaya, Aytekin Sönmeyenmakas, Pablo Prieto Gonzalez

**Affiliations:** 1 Faculty of Medicine, Tekirdağ Namık Kemal University, Tekirdağ, Türkiye; 2 Faculty of Sports Sciences, Tekirdağ Namık Kemal University, Tekirdağ, Türkiye; 3 Department of Rehabilitation Sciences, College of Health and Rehabilitation Sciences, Princess Nourah bint Abdulrahman University, P.O. Box 84428, Riyadh, 11671 Saudi Arabia; 4 Faculty of Sports Sciences, Istanbul Aydın University, Istanbul, Türkiye; 5 Faculty of Sports Sciences, Bandırma Onyedi Eylül University, Bandırma, Türkiye; 6 Centro de Investigação em Desporto, Educação Física, Exercício e Saúde (CIDEFES), Lusófona University, Porto, Portugal; 7 Faculty of Sports Sciences, Istanbul Aydın University, Istanbul, Türkiye; 8 Sport Sciences and Diagnostics Research Group, GSD-HPE Department, Prince Sultan University, Riyadh, Saudi Arabia

**Keywords:** Injury risk, Functional capacity, Mobility, Flexibility

## Abstract

The aim of the study was to determine the role of isometric strength and range of motion in predicting Functional Movement Screen (FMS) scores of adults. A total of 120 participants (age = 34.62 ± 11.82 years; height = 170.56 ± 9.63 cm; weight = 73.62 ± 15.39 kg) volunteered to participate in the study. Anthropometric measurements were performed, including height, body weight, muscle mass, and body fat. Following this, the ranges of motion of the shoulder, hip, knee, and ankle joints were measured sequentially. Isometric strength and FMS tests were then performed. Hip extension isometric strength explained 23% of the variation in FMS_total_. The common effect of knee flexion, shoulder flexion, and dorsiflexion joint range of motion explained 34% of the change in FMS_total_ (F _(3−116)_ = 20.375, *p* < 0.001). A significant relationship (*R* = 0.658, R^2^ = 0.413) was found between hip extension isometric strength, knee flexion, shoulder flexion, and dorsiflexion range of motion and FMS_total_ (F _(4−115)_ = 21.952, *p* < 0.001). The common effect of all these variables explains 43% of the change in FMS_total_. The results indicate that the FMS test scores, which are utilized to evaluate the risk of injury in sedentary adults, can be significantly predicted by the effect of hip extension isometric strength and parameters related to knee flexion, shoulder flexion, and dorsiflexion joint range of motion. At this time, it is advised that range of motion and isometric strength be taken into account when determining a person’s functional movement capacity.

## Introduction

Functional movement capacity is an essential component of general health and well-being and includes the capacity to carry out daily chores with efficiency and effectiveness [1]. Additionally, the graded and scored version of a person’s motions before executing a specified training regimen is known as functional movement capacity, also known as the ability to perform fundamental movement patterns [2]. It is known that there are different tools that evaluate fundamental movement patterns. The FMS is a popular battery of tests purported to assess trunk and core strength as well as the fundamental movement parameters [3]. FMS has now reached the importance of scientific attention and is recommended as an analysis tool to assess movement asymmetries and movement sample limitations dynamically and practically [4–6]. When it comes to predicting injuries or performance or assessing movement, the FMS is an effective instrument for coaches, trainers, and physical therapists due to these features [7].

The FMS, developed by Gray Cook and Lee Burton in 1997, is designed to identify movement deficiencies and predict injury risk through seven fundamental movement patterns. These patterns—deep squat, hurdle step, in-line lunge, shoulder mobility, active straight-leg raise, trunk stability push-up, and rotary stability—were used to investigate the relationship with range of motion (ROM) and isometric strength, which are critical for functional movement capacity.

In recent years, there has been a growing interest in understanding the complex interplay between various physical parameters and their impact on functional movement capacity in adults [3, 8]. In the literature, some studies have conducted the FMS test on athletes [9–11], sedentary individuals [12], elderly [13], children [14], and young people [15]. However, no study was found in which isometric strength and range of motion were evaluated to predict functional movement capacity. Understanding how isometric strength and range of motion affect functional movement capacity is paramount for several reasons. Firstly, it can provide valuable insights into the underlying mechanisms governing movement efficiency and quality. Secondly, it may inform the development of targeted interventions and exercise programs aimed at enhancing functional performance and mitigating movement-related impairments.

It has been reported that isometric strength, especially in individuals with high levels of force and explosive power, is strongly correlated with dynamic performance, one of the components assessed by FMS [16]. Isometric strength, the ability of a muscle or group of muscles to generate force without changing length, and range of motion, the extent of movement that a joint is capable of performing, are fundamental components of physical fitness and functionality [17, 18]. Particularly, having a strong core musculature, which is essential for the core strength evaluated in FMS, contributes to better results in FMS [19]. FMS scoring is based on the assessment of joint mobility and stability deficiencies, and a relationship between the joint range of motion (ROM) and FMS scoring has been demonstrated in university student-athletes [20]. On the other hand, due to high variations in ROM measurements in young and physically active individuals, the direction and validity of the relationship have not been fully expressed. It is believed that studies conducted in adults will provide more sensitive and valid results, and the impact of ROM on functional movement capacity will be more clearly observed. In our study, we aim to better understand how ROM affects functional movement capacity by selecting individuals with limited physical activity in an age range that better reflects the population.

We aimed to elucidate the extent to which isometric strength and range of motion influence functional movement capacity. Specifically, our objective was to ascertain whether functional parameters can predict an individual’s functional movement proficiency. Ultimately, our findings may have implications for optimizing movement strategies, promoting injury prevention, and fostering overall health and mobility in adults.

## Method

### Subjects

This is a cross-sectional study conducted with a sample of 120 voluntary adults (age = 34.62 ± 11.82 years; height = 170.56 ± 9.63 cm; weight = 73.62 ± 15.39 kg) recruited at the Tekirdağ Namık Kemal University. Ethical approval for the study was obtained from the Non-Invasive Clinical Research Ethics Committee of Tekirdağ Namık Kemal University (Approval number: 2023.37.02.15), and informed consent was obtained from all participants. The study aimed to examine the effects of range of motion (ROM) and isometric strength on functional movement capacity in a population representative of average adults who do not engage in regular physical activity. The inclusion criteria included being between the ages of 18 and 65, being physically independent, and not having any cardiac, orthopaedic, or musculoskeletal system dysfunctions. Additionally, participants did not engage in regular physical activity more than once a week in the five months prior to the start of the study. Exclusion criteria were chronic ankle instability and lower extremity musculoskeletal injury in the previous 6 months, those receiving hormonal replacement therapy, those with uncontrolled diabetes or hypertension. After fulfilling the inclusion criteria, participant consent forms were obtained before study entry, in accordance with the Helsinki Declaration and subsequent amendments [21]. Measurements were performed by an expert research team. During measurements, verbal and practical information was provided by the research team for each test parameter. Before the tests were conducted, participants were instructed to perform a 10-minute warm-up and stretching exercises. The warm-up consisted of calisthenic movements such as arm circles, hip circles, leg swings, jog in place, jumping jack, wall slides, hip rotations, body weight squat, supported lunge, skipping.

### Data collection

#### Body composition measurements

Participants’ height measurements were taken using the Mesilife 13,539 portable stadiometer (Istanbul, Türkiye). Participants stood barefoot with their feet together, knees straight, heels, buttocks, and scapulae in contact with the device, and with a straight posture in the Frankfort horizontal plane. Measurements were taken during the inhalation period of the deep breath [22]. Participant’s body weight, body fat percentage, and body mass index values were determined using a bioelectrical impedance analysis (BIA) device (Tanita, Tartı Fast, Japan). The BIA device, operating with a fixed current of 50 kHz and 8 electrodes (hand to hand, foot to foot), measured fat percentage, muscle mass, and fat-free mass values for five different regions (right and left arm, right and left leg, torso). Procedures followed the operational principles of the device [23]. Body mass index (BMI) was computed as kg/m^2^ [24].

### Physical performance tests

#### Range of motion

The Dualer IQ Pro Inclinometer (J-TECH Medical, Salt Lake City, USA) was used to measure participants’ joint range of motion. The device allows easy and reliable data collection through dual sensor measurement. The inclinometer measures the difference between two endpoints and determines the range of motion with a margin of error of 1 degree. Measurements were taken considering the average values set by the American Academy of Orthopaedic Surgeons (AAOS), which is one of the most commonly used criteria for normal joint motion measurement [25]. Shoulder flexion, extension, abduction, adduction, knee flexion, hip flexion, extension, hip abduction, hip abduction, adduction, dorsiflexion, plantar flexion ranges of motion were determined. A detailed example of the shoulder joint flexion and extension phase is presented below.

Shoulder Joint Flexion/Extension: The participant stands with the arm down and the shoulder in a neutral position. The first sensor is attached to the upper arm with a strap. The flexion value is taken by reaching the participant’s shoulder maximally forwards and extension values are taken by reaching backwards.

**Fig. 1:**
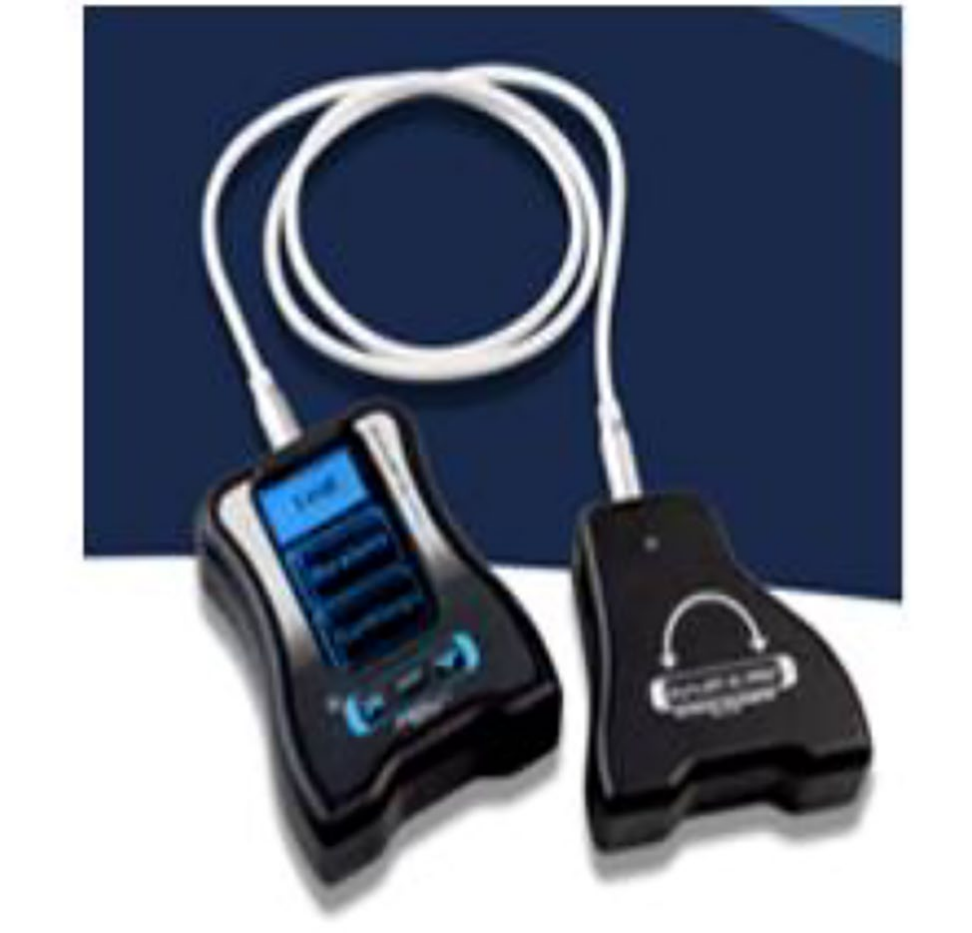
Inclinometer device

**Fig. 2:**
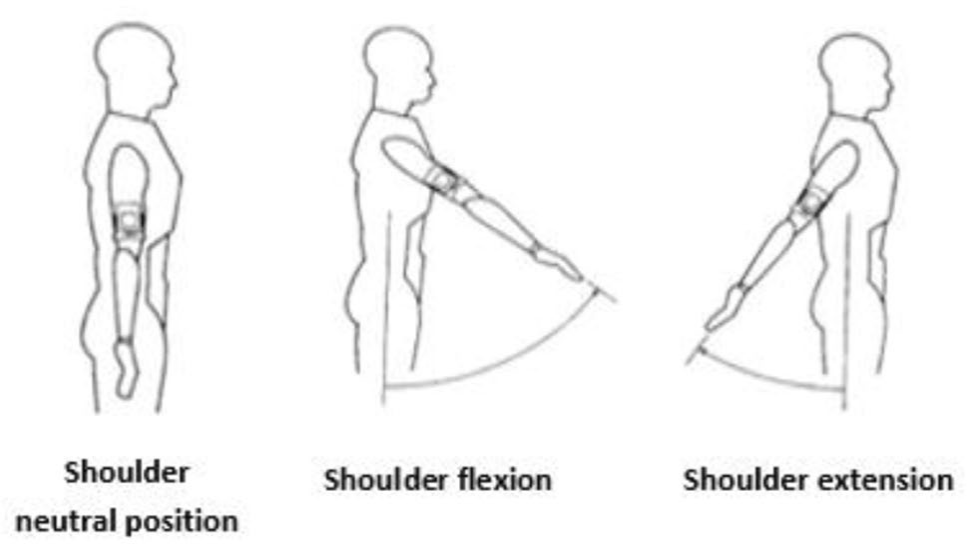
Shoulder flexion/extension sample

### Isometric muscle strength measurement protocol

The Lafayette Manual Muscle Test System, Model 01165 (Lafayette Instrument Company, Lafayette IN, USA), was used to determine participants’ isometric muscle strength. The Lafayette Manual Muscle Test (MMT) System is an ergonomic hand-held device used to objectively measure muscle strength. The test is performed by the clinician applying force to the patient’s limbs, to overcome or “break” the patient’s resistance. The MMT records the peak force and time required to achieve the “break,” providing reliable, accurate, and consistent muscle strength readings. The MMT also features interactive menus that allow for a variety of options, including data storage, preset test durations, and applied force thresholds. Its ergonomic design ensures compatibility with manual muscle testing protocols while providing comfort for both the patient and the testing device [26]. Shoulder flexion, extension, abduction, adduction, hip flexion, extension, abduction, adduction, knee flexion, and extension isometric force values were determined. A detailed example of knee flexion/extension muscle group isometric strength measurement is presented below.

Knee flexor muscle group isometric strength measurement: The participant is placed on the platform in the prone position. The participant is asked to bring the patellafemoral joint to a 90-degree angle to bring the movement to the starting position. The tester applies force with the dynamometer to make the hamstring extension. The participant resists the applied force and the value measured by the dynamometer is recorded [26].

Knee extensor muscle group isometric strength measurement: The participant sits on the platform with the patellofemoral joint at 90 degrees. The tester applies force to the participant’s lateral tibia with a dynamometer. The participant tries to resist the applied force. The value measured by the dynamometer is recorded [26].

**Fig. 3:**
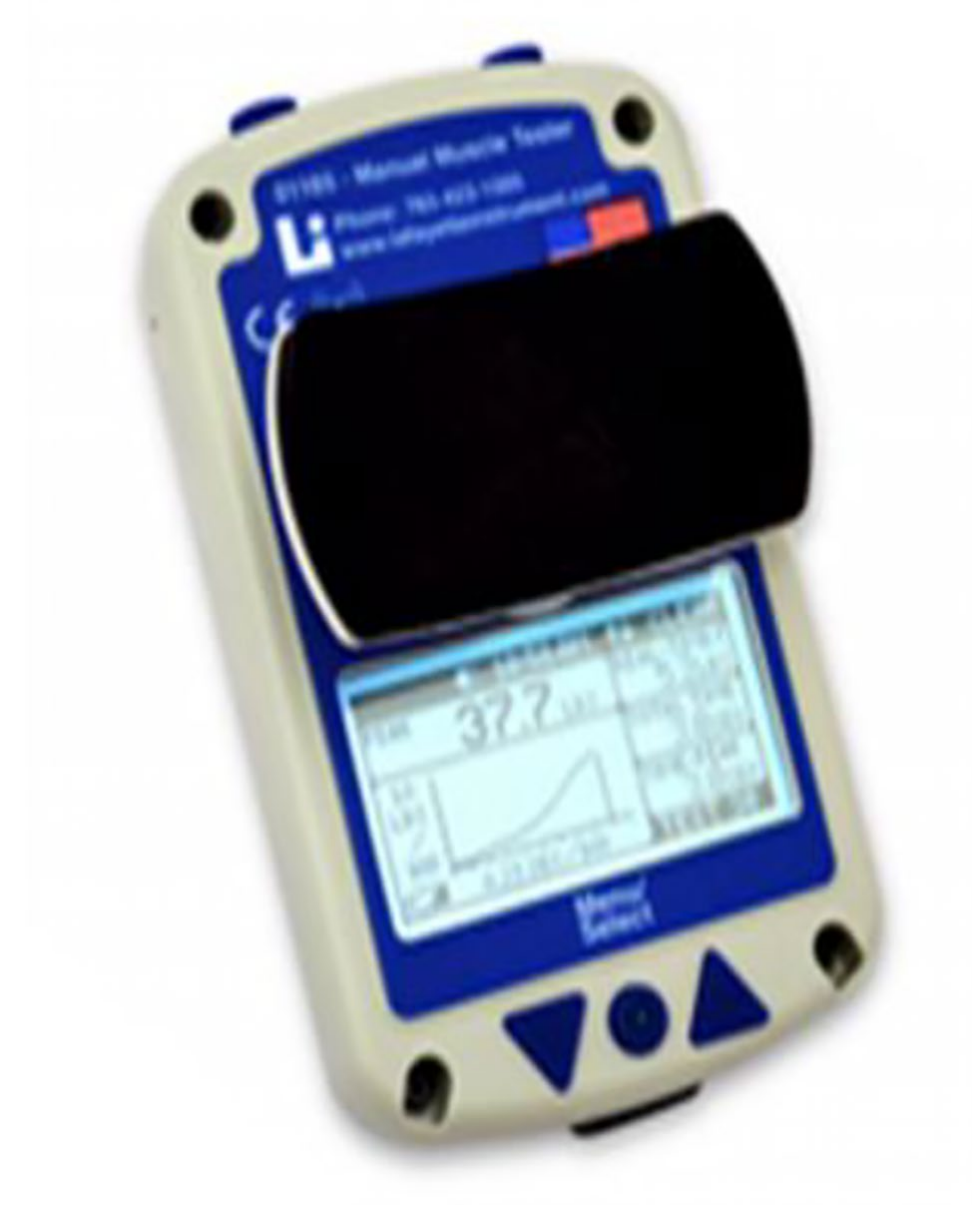
Manual muscle test device

**Fig. 4:**
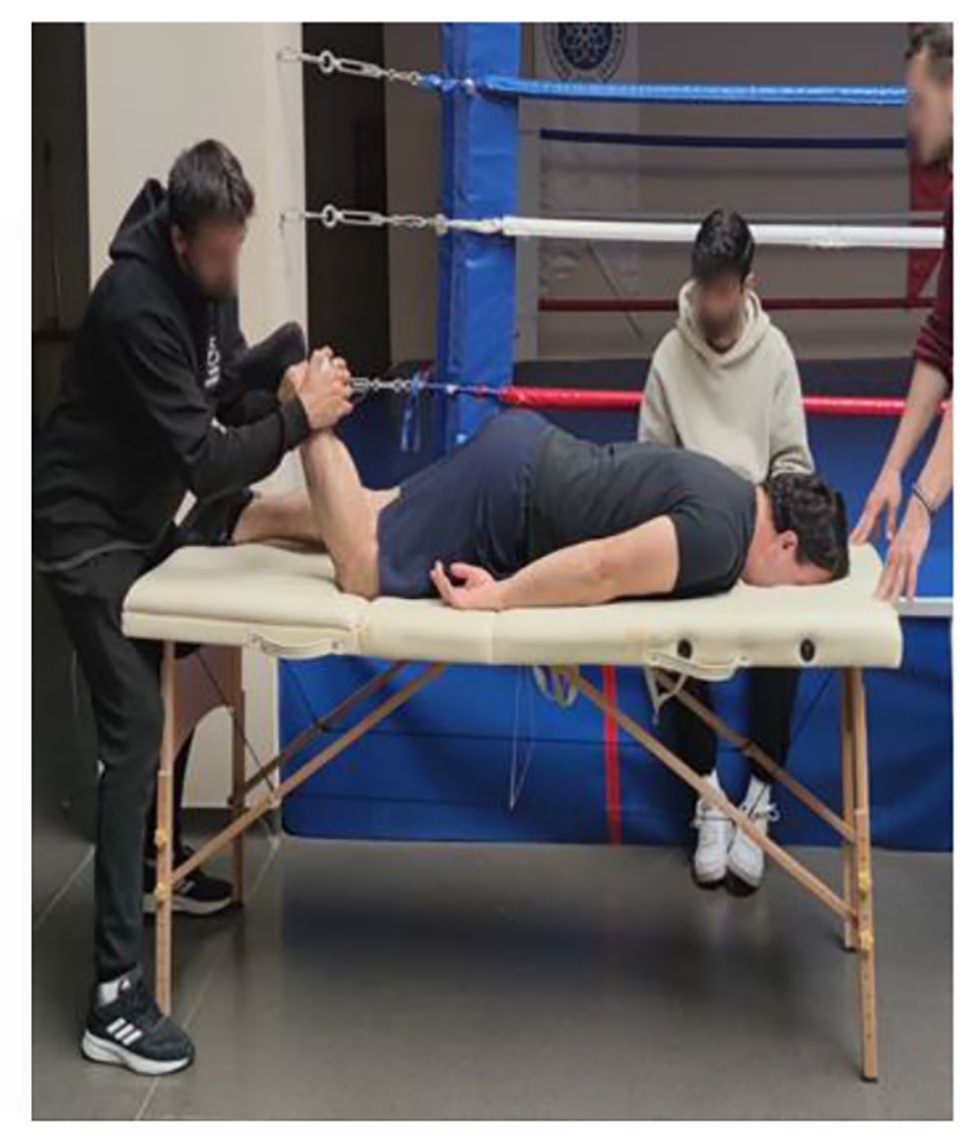
Knee flexion strength test

**Fig. 5:**
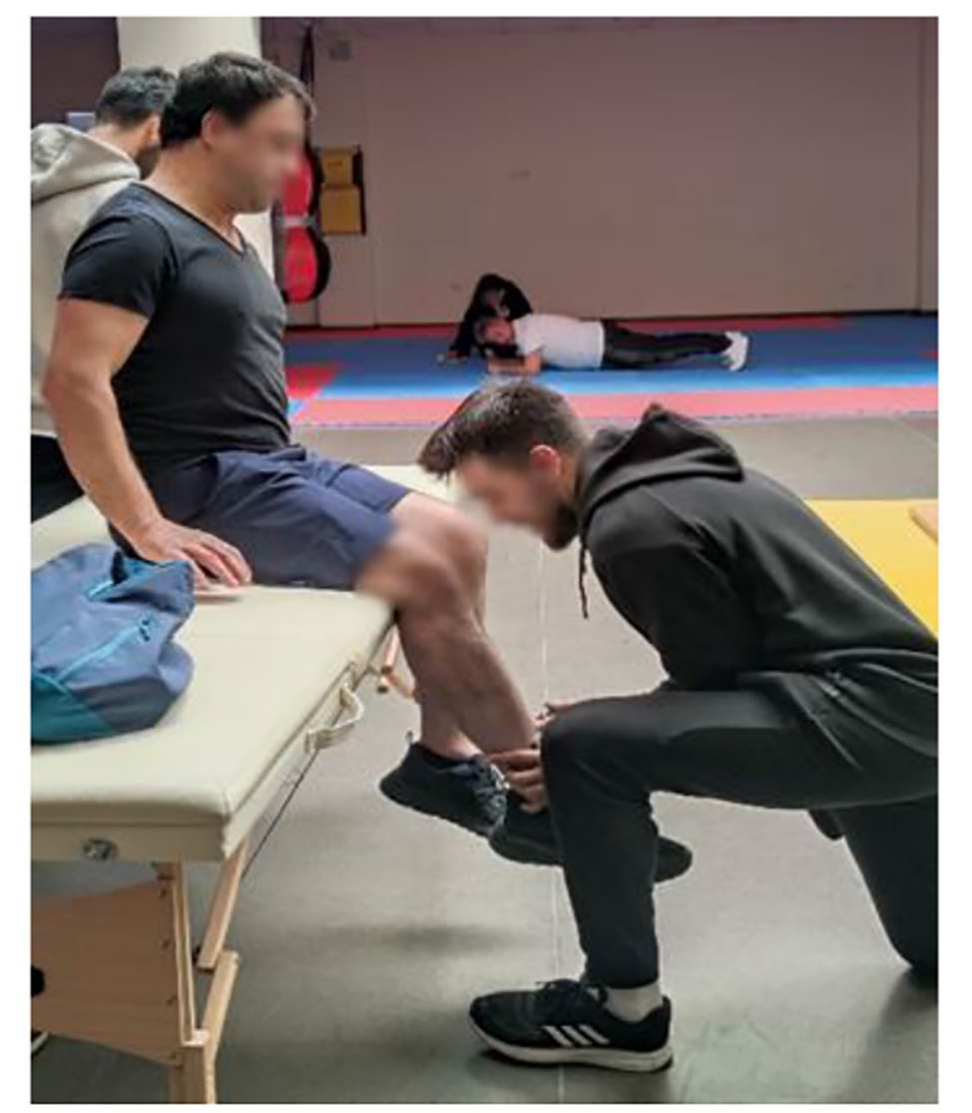
Knee extension strength test

### Functional movement screen test protocol

The Functional Movement Screen™ system, developed by Gray Cook, Lee Burton, and Keith Fields, is a system used to assess potential injury risks in athletes, determine the quality of individuals’ movement patterns, evaluate weaknesses in neuromuscular control, and enhance athletic performance [27, 28]. Conducted under the guidance of an expert, it is a screening system designed to allow an individual to assess their fundamental movement patterns. Such a screening system can also be a crucial tool in predicting injury, and determining readiness to return to sports after completing rehabilitation post-surgery, or during pre-participation evaluations [29]. The Functional Movement Screening test consists of 7 different basic movements (deep squat, hurdle step, single line step, shoulder mobility, active straight leg raises, trunk stability push-up, and rotation stability). Scoring for FMS consists of four different possibilities. Scores range from zero to three, with three being the best possible score. The maximum score for the FMS test is 21. Individuals scoring below 14 points may be at risk of disability [29].

### Procedure

**Fig. 6:**
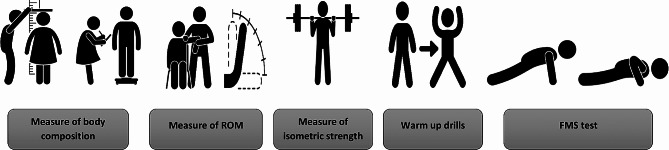
An illustrative summary diagram of the measurements performed in the study

The researchers provided theoretical and practical explanations of the test and measurement protocols to the participants. On the day of the tests, anthropometric measurements were performed, including height, body weight, muscle mass, and body fat. Following this, the range of motion was measured sequentially. Isometric strength and FMS tests were then performed. The tests were administered to the participants in the same order and by the same investigators. Before the FMS was assessed, a standardized warm-up consisting of 5 min of running and 5 min of dynamic stretching was performed. All tests were performed at the same time of day (09:30 − 11:30) to minimize the influence of circadian rhythms on the results. After the tests were completed, participants were instructed to perform cool-down exercises. Measurements and their sequence are shown in detail in Fig. 6.

### Statistical analysis

The data of the participants in the study were presented through descriptive statistical analyses, reporting mean ± standard deviation (SD) and frequency. We performed a priori sample size calculation using “pwr” R package. We performed an analysis using the f^2^ = 0.10, α = 0.05 and β = 0.10 (1-β = 0.90 power), which showed that a minimum sample of 108 subjects would be required. The assumption of normality of the variables was determined by the Shapiro-Wilk test, and the homogeneity of variance was determined by Mauchly’s Sphericity test. The significance level was accepted as 0.05 in the analyses. Additionally, multiple linear regression analysis was conducted to examine the effect of ROM and isometric strength parameters on FMS_total_ score within the scope of the study’s objectives. Both stepwise and enter models were employed. The primary dependent variable in our regression models was functional movement capacity, as measured by the Functional Movement Screen (FMS) total score. The independent variables included: ROM measurements for various joints (shoulder, knee, hip, and ankle), and isometric strength measurements for specific muscle groups. We employed a stepwise regression approach to identify the most significant predictors of functional movement capacity. The stepwise process involved creating an initial model, selecting variables based on their significance, and adding them one by one until no significant improvements were observed in the model fit. The criterion for inclusion was a p-value < 0.05, while the criterion for exclusion was a p-value > 0.10.

## Results

Mean and standard deviation values for age and anthropometric parameters are shown in Table 1 with distribution by sex and percentages of participants.

**Table 1:** Descriptive data on participants’ age and gender, body composition

Parameters	Male (Mean ± SD)	Female (Mean ± SD)	Total Mean ± SD
Age (years)	30.31 ± 11.44	40.07 ± 9.98	34.62 ± 11.82
Height (cm)	176.13 ± 6.71	163.52 ± 8.03	170.56 ± 9.63
Weight (kg)	79.21 ± 15.77	66.55 ± 11.63	73.62 ± 15.39
BMI (kg/m^2^)	25.82 ± 4.79	25.00 ± 4.88	25.45 ± 4.83
Body Fat (%)	21.02 ± 8.73	29.46 ± 8.53	24.75 ± 9.58
Muscle Mass (kg)	59.01 ± 6.29	43.91 ± 5.45	52.34 ± 9.57
Gender	**n / %**		
Male	67 / 55.8		
Female	53 / 44.2		

SD: Standard Deviation

The mean and standard deviation values of isometric strength, ROM parameters and FMS parameters of the participants are detailed in Table 2.

**Table 2:** Descriptive data on participants’ isometric strength, ROM, and FMS values

Isometric strength parameters (peak)	Mean ± SD	ROM parameters	Mean ± SD	FMS parameters	Mean ± SD
Shoulder flexion (lb)	20.87 ± 7.96	Shoulder flexion °	170.00 ± 14.09	Deep squat	2.30 ± 0.73
Shoulder extension (lb)	16.38 ± 6.20	Shoulder extension °	61.80 ± 18.60	Hurdle step	2.36 ± 0.55
Shoulder abduction (lb)	18.45 ± 6.74	Shoulder abduction °	174.92 ± 20.50	Inline lunge	2.20 ± 0.74
Shoulder adduction (lb)	15.72 ± 5.67	Shoulder adduction °	25.01 ± 14.18	Shoulder mobility	2.48 ± 0.68
Hip flexion (lb)	23.65 ± 8.07	Knee flexion °	118.76 ± 17.23	Active straight-leg raise	2.45 ± 0.54
Hip extension (lb)	23.93 ± 9.48	Hip flexion °	105.13 ± 30.38	Trunk stability-push up	2.07 ± 0.91
Hip abduction (lb)	26.24 ± 9.11	Hip extension °	50.02 ± 26.74	Rotary stability	1.93 ± 0.57
Hip adduction (lb)	17.45 ± 6.84	Hip abduction °	32.20 ± 15.28	Total FMS Score	15.81 ± 3.06
Knee flexion (lb)	17.22 ± 6.74	Hip adduction °	32.85 ± 11.30		
Knee extension (lb)	20.23 ± 6.48	Dorsiflexion °	27.29 ± 7.06		
		Plantar flexion °	52.86 ± 10.90		

lb = libra pondo, pounds

From the results obtained from the stepwise model of multiple linear regression analysis, a significant relationship (*R* = 0.481, R^2^ = 0.232) was found between hip extension isometric strength and FMS_total_ (F_(1−117)_ = 35.307, *p* < 0.001). Hip extension isometric strength explained 23% of the variation in FMS_total_ (Table 3). Other isometric strength parameters were not included in the analysis as a result of the Stepwise model.

**Table 3:** The multiple linear regression analysis outcomes of isometric strength parameters predicting performance on FMS

FMS	Predictors	B	SE	β	t	*p*	*R*	*R* ^2^	Adj.*R*^2^
Model 1	(Constant)	12.035	0.683	-	17.615	< 0.001	-	-	-
	Hip extension	0.157	0.026	0.481	5.942	< 0.001	0.481	0.232	0.225

FMS Model 1: (F_(1−117)_ = 35.307, *p* < 0.001)

From the results obtained from the stepwise model of multiple linear regression analysis, the joint effect of knee flexion, shoulder flexion, and ankle dorsiflexion joint range of motion explained 34% of the change in FMS_total_ (F _(3−116)_ = 20.375, *p* < 0.001). In addition, a significant relationship (*R* = 0.587, R^2^ = 0.345) between these variables and FMS_total_ was determined (Table 4). Furthermore, other ranges of motion parameters were not included in the analysis as a result of the stepwise model.

**Table 4:** The multiple linear regression analysis outcomes of range of motion parameters predicting performance on FMS

FMS	Predictors	B	SE	β	t	*p*	*R*	*R* ^2^	Adj.*R*^2^
Model 1	(Constant)	6.999	1.787	-	3.916	< 0.001	0.417	0.174	0.167
	Knee flexion	0.074	0.015	0.417	4.985	< 0.001			
Model 2	(Constant)	-3.958	3.161	-	-1.252	0.213	0.527	0.277	0.265
	Knee flexion	0.065	0.014	0.365	4.588	< 0.001			
	Shoulder flexion	0.071	0.017	0.326	4.091	< 0.001			
Model 3	(Constant)	-5.840	3.070	-	-1.902	0.060	0.587	0.345	0.328
	Knee flexion	0.063	0.014	0.354	4.654	< 0.001			
	Shoulder flexion	0.065	0.017	0.299	3.904	< 0.001			
	Dorsiflexion	0.114	0.033	0.262	3.464	0.001			

**FMS Model 1**: (F_(1−118)_ = 24.854, *p* < 0.001); **FMS Model 2**: (F_(2−117)_ = 22.453, *p* < 0.001)

**FMS Model 3**: (F_(3−116)_ = 20.375, *p* < 0.001)

From the results obtained from the enter model of multiple linear regression analysis, a significant relationship (*R* = 0.658, R^2^ = 0.413) was found between hip extension isometric strength, knee flexion, shoulder flexion, and dorsiflexion range of motion and FMS_total_ (F_(4−115)_ = 21.952, *p* < 0.001). The joint effect of all these variables explains 43% of the change in FMS_total_ (Table 5).

**Table 5:** The multiple linear regression analysis outcomes of hip extension isometric strength, knee flexion, shoulder flexion and dorsiflexion range of motion predicting performance on FMS

FMS	Predictors	B	SE	β	t	*p*	*R*	*R* ^2^	Adj.*R*^2^
Model 1	(Constant)	-3.877	2.907	-	-1.334	0.185	0.658	0.433	0.413
	Hip extension	0.103	0.024	0.317	4.221	< 0.001			
	Knee flexion	0.051	0.013	0.287	3.930	< 0.001			
	Shoulder flexion	0.049	0.016	0.224	3.049	0.003			
	Dorsi flexion	0.105	0.031	0.242	3.412	<0.001			

**FMS Model 1**: (F_(4−115)_ = 21.952, *p* < 0.001)

## Discussion

The aim of the study was to determine the predictability of FMS scores evaluating functional movement capacity in terms of range of motion and isometric strength in healthy adults. The study revealed that hip extension isometric strength predicted FMS total scores by 23%. The joint effect of knee flexion, shoulder flexion, and dorsiflexion on FMS total scores was found to be 34%. However, hip extension isometric force, knee flexion, shoulder flexion, and dorsiflexion predicted FMS total scores by 43%.

Low FMS_total_ are, at least partially, a function of movement deficits [3, 30] and have been linked to injury [31, 32]. Muscle strength is one of the most fundamental physical elements, associated with the quality of movement creation and execution in daily physical activities and athletic performance. Measuring and classifying strength levels are crucial for making decisions in physical exercise prescription and treatment [33]. Understanding isometric strength and its correlation with functional movement capacity can aid in developing a training programme that effectively reduces the likelihood of injury in sedentary individuals as well as athletes. In a study conducted on children, a small correlation was found between core strength and FMS scores [34]. Okada et al. [19] who found no correlation between these 2 variables in healthy college-aged adults. Additionally, a growing amount of research suggests that traits related to the foot and ankle may have an impact on how well a person does on balance and functional tests [35]. It has been demonstrated that the functional movements of stooping, crouching, and kneeling are connected with the strength of the ankle dorsiflexor and plantar flexor muscles [36]. These functional movements, while not deliberately tested, are required for adequate completion of FMS tasks and are indirectly assessed within the context of the FMS test battery. These movements require adequate ankle mobility and stability, which are influenced by the strength of the ankle dorsiflexors and plantar flexors. Therefore, while the FMS does not include specific tests for stooping, crouching, and kneeling, aspects of ankle strength and mobility are indirectly assessed within the context of the FMS test battery. A prior study found a substantial correlation between balance and functional capacity and the strength of the toe plantar flexors, ankle dorsiflexion range of motion, foot posture, and the existence of hallux valgus [37]. The most reliable, significant, and independent predictors of balance and functional test performance were found to be hallux plantar flexion strength and ankle inversion-eversion range of motion in a cross-sectional study of adults over 65. These predictors could account for up to 25% of the variance in test scores [35].

In the prior studies for the squat task, it was found that higher FMS squat and lunge task scores were generally associated with more ankle dorsiflexion ROM [20, 38–40]. Determining the joint range of motion allows physicians and physiotherapists to diagnose musculoskeletal function, monitor the progress of an intervention, record data for future follow-ups, and meet legal requirements for impairment ratings and injury determinations when applicable [41]. Additionally, it plays a significant role in sports science for assessing athletic and sedentary performance and determining physical performance when preparing individualized exercise programs [42].

Hincapié et al. [20] found that athletes with the highest hip extension ROM tended to be those with the highest lunge scores, while other studies differed from previous findings [43, 44]. In our study, it was determined that there was a relationship between FMS total scores and shoulder flexion range of motion. The results of the study by Hincapié et al. support our study. Hincapié et al. [20] determined that shoulder flexion ROM was generally greater in those athletes with higher shoulder and squat task scores. However, previous research failed to find relationships between shoulder mobility test scores and glenohumeral joint ROM measurements [45]. Excessive mobility of the shoulder complex can compromise joint stabilisation, leading to conditions such as shoulder dislocation, which can cause damage to the constituent elements of the shoulder joint structure. [20]. Apart from this, some studies reveal how strength and ROM variables affect individuals’ activities of daily living. Reduced lower-extremity range of motion (ROM) was linked, according to Bergstrom and colleagues [46], to self-reported difficulties with functional mobility, including getting out of a chair, mounting stairs, and requiring assistive devices when walking. According to Woolley and colleagues [47], in subjects with osteoarthritis, knee extension force and subject pain rating during the floor rise accounted for 28% of the variability in timed completion of this task. According to Woolley et al. [47], knee flexion and extension force, body weight, and reported function all accounted for 47% of the variation in stair ascending time. Other researchers discovered that the minimal chair height that a person can rise from [48] and the rate at which a person may rise from a chair [49] are both determined by lower-extremity force.

### Strength and limitation

The strength of the study is that isometric strength and range of motion parameters were considered together and the effect on FMS scores was revealed.

The most important limitation of the study is that the information about the participants’ exercise or sports history was not determined, and in addition, their current physical activity status was not determined during the measurement process. Our study included participants who reported engaging in regular physical activity less than one day per week. This criterion was established to focus on a population with minimal physical activity levels, allowing us to assess the impact of range of motion (ROM) and isometric strength on functional movement capacity in individuals who are less physically active. While this selection criterion provided valuable insights into the relationships studied, it is important to acknowledge that the findings may not directly generalize to more active populations. One of the main limitations of our study is the lack of detailed reliability testing for the isometric strength measures used. Although we employed standardized protocols and trained evaluators to ensure consistency, a comprehensive reliability analysis including intra-rater and inter-rater reliability assessments was not conducted. Future studies should prioritize rigorous reliability testing to enhance the validity and generalizability of the findings related to isometric strength.

## Conclusion

This study highlights the critical role of specific physical attributes in functional movement capacity. Notably, hip extension isometric strength emerged as a key predictor of overall FMS performance, underscoring its importance in movement quality. Additionally, range of motion in knee flexion, shoulder flexion, and dorsiflexion significantly contributed to functional movement scores.

These findings have practical implications for both clinical and athletic settings. For practitioners, focusing on enhancing hip extension strength and improving flexibility in key joints can lead to better movement efficiency and reduced injury risk. Fitness professionals and coaches can incorporate targeted strength and flexibility exercises into training programs to optimize functional movement and performance outcomes.

In summary, developing targeted interventions to improve hip extension strength and joint flexibility can significantly enhance functional movement capacity, providing a clear pathway for improving overall physical health and performance.

## Data Availability

The datasets generated and/or analysed during the current study are not publicly available but are available from the corresponding author on reasonable request.

## References

[1] Frost DM, Beach TA, Callaghan JP, McGill SM (2012). Using the Functional Movement Screen™ to evaluate the effectiveness of training. J Strength Cond Res.

[2] Vehrs PR, Uvacsek M, Johnson AW (2021). Assessment of dysfunctional movements and asymmetries in children and adolescents using the Functional Movement Screen—A narrative review. Int J Environ Res Public Health.

[3] Cook G, Burton L, Hoogenboom B (2006). Pre-participation screening: the use of fundamental movements as an assessment of function–part 1. Int J Sports Phys Ther.

[4] Minthorn LM, Fayson SD, Stobierski LM, Welch CE, Anderson BE (2015). The Functional Movement screen’s ability to detect changes in movement patterns after a training intervention. J Sport Rehabil.

[5] Kraus K, Schütz E, Taylor WR, Doyscher R (2014). Efficacy of the functional movement screen: a review. J Strength Cond Res.

[6] Dorrel BS, Long T, Shaffer S, Myer GD (2015). Evaluation of the functional movement screen as an injury prediction tool among active adult populations: a systematic review and meta-analysis. Sports Health.

[7] Girard J, Quigley M, Helfst F (2016). Does the functional movement screen correlate with athletic performance? A systematic review. Phys Ther Rev.

[8] Hrysomallis C, Hopkins G (2015). Protective headgear for rugby, Australian rules football and soccer. Sports injuries: Prevention, Management and Risk factors.

[9] Garrison M, Westrick R, Johnson MR, Benenson J (2015). Association between the functional movement screen and injury development in college athletes. Int J Sports Phys Ther.

[10] Kiesel KB, Butler RJ, Plisky PJ (2014). Prediction of injury by limited and asymmetrical fundamental movement patterns in American football players. J Sport Rehabil.

[11] Azzam MG, Throckmorton TW, Smith RA, Graham D, Scholler J, Azar FM (2015). The Functional Movement Screen as a predictor of injury in professional basketball players. Curr Orthop Pract.

[12] Hanlon M. Assessing the validity and test retest reliability of the Kinect sensor when scoring the functional movement screen. In: European College of Sports Science Conference. Essen, Germany; 2017.

[13] Loudon JK, Parkerson-Mitchell AJ, Hildebrand LD, Teague C (2014). Functional movement screen scores in a group of running athletes. J Strength Cond Res.

[14] Parenteau -GE, Gaudreault N, Chambers S, Boisvert C, Grenier A, Gagné G (2014). Functional movement screen test: a reliable screening test for young elite ice hockey players. Phys Ther Sport.

[15] Everard EM, Harrison AJ, Lyons M (2017). Examining the relationship between the functional movement screen and the landing error scoring system in an active, male collegiate population. J Strength Cond Res.

[16] Juneja H, Verma S, Khanna G (2012). Isometric strength and its relationship to dynamic performance: a systematic review. J Exerc Sci Physiotherapy.

[17] Haff GG, Triplett NT. Essentials of strength training and conditioning. 4th ed. Human kinetics; 2015.

[18] Wilk M, Golas A, Stastny P, Nawrocka M, Krzysztofik M, Zajac A (2018). Does tempo of resistance exercise impact training volume?. J Hum Kinet.

[19] Okada T, Huxel KC, Nesser TW (2011). Relationship between core stability, functional movement, and performance. J Strength Conditioning Res.

[20] Hincapié CA, Tomlinson GA, Hapuarachchi M, Stankovic T, Hirsch S, Carnegie D (2022). Functional Movement Screen task scores and joint range-of-motion: a construct validity study. Int J Sports Med.

[21] Association WM (2013). World Medical Association Declaration of Helsinki: ethical principles for medical research involving human subjects. JAMA.

[22] Lohman TG (1988). Anthropometric standardization reference manual.

[23] Saritas N, Ozkarafaki I, Pepe O, Buyukipekci S. Evaluation of body fat percentage of female university students according to three different methods. In: 11th International Scientific Conference Perspectives In Physical Education and Sport. vol. 11. Constanta, Romania; 2011: 244-9.

[24] Status WECP. The Use of and Reading of Anthropometry, Report by a WHO Expert Committee. In: WHO Technical Report Series. World Health Organization; 1995. 8594834

[25] Surgeons AAoO (1965). Joint motion: method of measuring and recording.

[26] Instrument L. The Lafayette Manual Muscle Test System User’s Manual. In. Edited by Company LI; 2012: 1–20.

[27] Clark SC, Rowe ND, Adnan M, Brown SM, Mulcahey MK (2022). Effective Interventions for Improving Functional Movement Screen scores among high-risk athletes: a systematic review. Int J Sports Phys Ther.

[28] Doyscher R, Schütz E, Kraus K (2016). Evidence of the Functional Movement Screen in high-level sports - a structured review with own data. Sport Orthop Traumatol.

[29] Cook G, Burton L, Hoogenboom BJ, Voight M (2014). Functional movement screening: the use of fundamental movements as an assessment of function-part 1. Int J Sports Phys Ther.

[30] Cook G, Burton L, Torine J, Movement. Functional movement systems: Screening, assessment and corrective strategies. 2010.

[31] Kiesel K, Plisky PJ, Voight ML (2007). Can serious injury in professional football be predicted by a preseason functional movement screen?. N Am J Sports Phys Ther.

[32] O’connor FG, Deuster PA, Davis J, Pappas CG, Knapik JJ (2011). Functional movement screening: predicting injuries in officer candidates. Med Sci Sports Exerc.

[33] Wikholm JB, Bohannon RW (1991). Hand-held dynamometer measurements: tester strength makes a difference. J Orthop Sports Phys Ther.

[34] Mitchell UH, Johnson AW, Adamson B (2015). Relationship between functional movement screen scores, core strength, posture, and body mass index in school children in Moldova. J Strength Cond Res.

[35] Spink MJ, Fotoohabadi MR, Wee E, Hill KD, Lord SR, Menz HB (2011). Foot and ankle strength, range of motion, posture, and deformity are associated with balance and functional ability in older adults. Arch Phys Med Rehabil.

[36] Hernandez ME, Goldberg A, Alexander NB (2010). Decreased muscle strength relates to self-reported stooping, crouching, or kneeling difficulty in older adults. Phys Ther.

[37] Menz HB, Morris ME, Lord SR (2005). Foot and ankle characteristics associated with impaired balance and functional ability in older people. J Gerontol Biol Sci Med Sci.

[38] Chimera NJ, Knoeller S, Cooper R, Kothe N, Smith C, Warren M (2017). Prediction of functional movement screen™ performance from lower extremity range of motion and core tests. Int J Sports Phys Ther.

[39] Rabin A, Kozol Z (2017). Utility of the overhead squat and forward arm squat in screening for limited ankle dorsiflexion. J Strength Conditioning Res.

[40] Gomes J, Neto T, Vaz JR, Schoenfeld BJ, Freitas SR (2022). Is there a relationship between back squat depth, ankle flexibility, and Achilles tendon stiffness?. Sports Biomech.

[41] Youdas JW, Carey JR, Garrett TR (1991). Reliability of measurements of cervical spine range of motion—comparison of three methods. Phys Ther.

[42] Senturk A, Kuyulu İ, Zorba E (2022). Eklem Hareket Genişliği Ölçüm Yöntemleri ve Yüzme Performansı Üzerine Etkileri. Spor Bilimlerinde Akademik Çalışmalar-18.

[43] Frost D, Andersen J, Lam T, Finlay T, Darby K, McGill S (2013). The relationship between general measures of fitness, passive range of motion and whole-body movement quality. Ergonomics.

[44] Noda T, Verscheure S (2009). Individual goniometric measurements correlated with observations of the deep overhead squat. Athletic Train Sports Health Care.

[45] Sprague PA, Mokha GM, Gatens DR, Rodriguez R (2014). The relationship between glenohumeral joint total rotational range of motion and the functional movement screen™ shoulder mobility test. Int J Sports Phys Ther.

[46] Bergström G, Aniansson A, Bjelle A, Grimby G, Lundgren-Lindquist B, Svanborg A (1985). Functional consequences of joint impairment at age 79. Scand J Rehabil Med.

[47] Woolley S, Topp R, Khuder S, Kahaleh B, Commager J. Function: which factors predict ability in OA patients. Biomech Geriatr. 1998:6–12.

[48] Hughes MA, Myers BS, Schenkman ML (1996). The role of strength in rising from a chair in the functionally impaired elderly. J Biomech.

[49] Bassey E, Bendall M, Pearson M (1988). Muscle strength in the triceps surae and objectively measured customary walking activity in men and women over 65 years of age. Clin Sci (Lond).

